# Development of a Clinical Risk Assessment Score for Respiratory Distress Due to Respiratory Infections in Early Infancy

**DOI:** 10.3390/children12060746

**Published:** 2025-06-09

**Authors:** Cristina Elena Singer, Cristina Popescu, Diana-Maria Trasca, Renata-Maria Varut, Rebecca-Cristiana Serban, Jaqueline Abdul-Razzak, Virginia-Maria Radulescu

**Affiliations:** 1 Department of Mother and Baby, University of Medicine and Pharmacy of Craiova, 200349 Craiova, Romania; cristina.singer@umfcv.ro (C.E.S.); jaquelineabdulrazzak90@gmail.com (J.A.-R.); 2 Department of Anatomy, University of Medicine and Pharmacy, Discipline of Anatomy, 200349 Craiova, Romania; cristina.popescu@umfcv.ro; 3 Department of Internal Medicine, University of Medicine and Pharmacy of Craiova, 200349 Craiova, Romania; 4 Research Methodology Department, Faculty of Pharmacy, University of Medicine and Pharmacy of Craiova, 200349 Craiova, Romania; 5 Cellular and Molecular Biology, University of Medicine and Pharmacy, Discipline of Anatomy, 200349 Craiova, Romania; drrebecastefan@yahoo.com; 6 Department of Medical Informatics and Biostatistics, University of Medicine and Pharmacy of Craiova, 200349 Craiova, Romania; virginia.radulescu@umfcv.ro

**Keywords:** neonatal respiratory distress, risk stratification, N-CRAS, prematurity, clinical severity prediction

## Abstract

Background/Objectives: Neonatal and infant respiratory distress carries high morbidity, and severity can vary with gestational maturity and perinatal factors. Early risk stratification may improve outcomes, but existing assessment tools do not fully address age-related risk differences. We aimed to develop and validate a Neonatal Clinical Risk Assessment Score (N-CRAS) for predicting severity in neonates and young infants with respiratory distress due to respiratory infection. Methods: This pilot score was applied exclusively to a cohort of forty neonates and young infants with respiratory distress secondary to infectious causes, as defined by clinical and laboratory parameters. Clinical variables (gestational age, delivery mode, birth weight category, and APGAR score) were recorded and analyzed for associations with illness severity. We developed the N-CRAS (0–5 points) encompassing five indicators of severe illness (respiratory infection, metabolic disorder, need for symptomatic treatment, mechanical ventilation, and intubation), each contributing 1 point. Patients were stratified as low (0–1), moderate (2–3), or high (4–5) risk. Chi-square tests and Spearman correlation assessed associations, and an ROC curve evaluated the score’s predictive performance for intubation. Results: No individual perinatal factor was significantly associated with respiratory illness severity. The N-CRAS increased with infant age (*p* < 0.05), indicating older infants tended to have more severe disease. All study infants who required intubation fell into the high-risk category (score ≥ 4). The N-CRAS demonstrated excellent discrimination for predicting intubation (ROC area under the curve = 1.00). Conclusions: In this pilot study, the N-CRAS demonstrated a strong correlation with clinical severity and successfully identified all infants who required intubation. However, given the small cohort size and limited number of severe cases, these findings should be interpreted cautiously. Further external validation in larger and more diverse neonatal populations is essential to confirm its predictive utility.

## 1. Introduction

Neonatal respiratory disorders constitute a major clinical challenge in modern medicine and are among the leading causes of morbidity and mortality in newborns. During the neonatal period, the respiratory system is uniquely vulnerable, and a variety of conditions may compromise breathing, ranging from intrinsic lung immaturity to infections and aspiration events. Conditions such as respiratory distress syndrome, transient tachypnea of the newborn, neonatal pneumonia, and meconium aspiration syndrome are encountered frequently in clinical practice. These disorders not only impose a significant burden on healthcare resources but also profoundly impact long-term outcomes. The clinical management of these infants is further complicated by the heterogeneous nature of their presentations and the myriad factors that influence disease severity [1,2,3,4,5].

The complexity of neonatal respiratory pathology is underscored by the fact that the signs of distress—tachypnea, retractions, nasal flaring, grunting, and cyanosis—are often nonspecific, requiring careful evaluation to distinguish between pulmonary and non-pulmonary causes. For instance, while a preterm infant may develop respiratory distress as a direct consequence of surfactant deficiency, a full-term neonate might exhibit similar symptoms due to an acquired infection or meconium aspiration. Such variability necessitates a thorough assessment that takes into account not only the immediate clinical findings but also the context in which these symptoms occur [6,7,8,9,10].

A critical factor in the evaluation of neonatal respiratory disorders is the age at which symptoms manifest. In the immediate postnatal period, respiratory difficulties are frequently related to perinatal events or intrinsic developmental factors, whereas later onset of respiratory compromise may signal the emergence of infections or other acquired conditions. Premature infants are particularly at risk, as their immature lungs predispose them to conditions like respiratory distress syndrome, which tend to be more severe and are associated with higher mortality rates. Conversely, in term infants, conditions such as transient tachypnea of the newborn or meconium aspiration syndrome predominate, reflecting a different underlying pathophysiology. The timing of symptom onset therefore provides valuable clues regarding the etiology of respiratory distress and guides clinicians in selecting appropriate diagnostic and therapeutic interventions [11,12,13,14].

Given the diverse causes of neonatal respiratory distress, risk stratification becomes a vital tool for optimizing care. Age-related risk stratification offers a structured approach to categorizing infants based on the timing and severity of their symptoms. It allows clinicians to identify those neonates and young infants who are most likely to experience a severe clinical course and who might benefit from early, aggressive intervention. In many neonatal intensive care units, clinical decisions are often based on a combination of physiological parameters and heuristic judgment; however, there is a growing recognition that standardized risk scores can improve decision making by providing an objective measure of illness severity [15,16].

Several scoring systems have been developed to assess the overall health status of study infants, yet most are focused on general indicators of critical illness rather than on specific aspects of respiratory failure. Tools such as the Apgar score, although useful at birth, offer only a snapshot of the newborn’s condition and do not capture the dynamic evolution of respiratory pathology over time. More comprehensive scoring systems have been introduced in the neonatal setting, but these tend to emphasize overall morbidity or mortality risk rather than the nuances of respiratory distress. There is, therefore, a clear need for a clinical tool that integrates the age of the patient—a critical determinant of respiratory pathology—with other key clinical variables to provide a tailored risk assessment for neonates and young infants with respiratory disorders [17,18,19].

It is within this context that the Neonatal Clinical Risk Assessment Score (N-CRAS) was developed. The N-CRAS was designed to be a simple yet robust tool for early risk stratification based on the clinical characteristics that are most predictive of severe respiratory outcomes. By incorporating variables such as the presence of respiratory infections, metabolic disturbances, and the need for supportive measures like mechanical ventilation and intubation, the N-CRAS aims to stratify neonates and young infants into risk categories that can inform clinical decision making. The goal is to identify those infants who are most at risk for deterioration and who may require more intensive monitoring or intervention [20,21].

The development of the N-CRAS reflects a data-driven approach to risk assessment. In clinical practice, the rapid identification of high-risk neonates and young infants can be critical in guiding therapeutic decisions, optimizing resource allocation, and ultimately improving outcomes. For instance, neonates and young infants with a high N-CRAS might be prioritized for advanced respiratory support or early initiation of therapies targeted at mitigating lung injury. Moreover, by quantifying risk at an early stage, the N-CRAS provides an objective benchmark that can be used in future research to evaluate the effectiveness of novel therapeutic strategies and to standardize comparisons across different clinical settings [22,23].

In addition to its potential clinical utility, the N-CRAS also contributes to our understanding of the interplay between age and respiratory pathology. Neonatal respiratory disorders do not occur in isolation; they are influenced by a host of factors, including gestational maturity, perinatal history, and the timing of symptom onset. By explicitly incorporating age into the risk stratification process, the N-CRAS acknowledges that the natural history of respiratory distress can vary significantly across different postnatal time points. This, in turn, emphasizes the need for age-specific treatment protocols and underscores the importance of tailoring interventions to the individual needs of the patient. Furthermore, the introduction of an age-related risk stratification tool like the N-CRAS represents a step toward personalized medicine in neonatology. In an era where precision medicine is increasingly becoming the standard, having a simple yet effective scoring system that captures the complexity of neonatal respiratory pathology is of paramount importance. Such a tool not only assists clinicians in making more informed decisions but also serves as a valuable adjunct in clinical research, facilitating the evaluation of new interventions and the refinement of treatment protocols [24,25,26,27].

The development and validation of the N-CRAS were driven by the observation that traditional risk assessment methods often fall short in capturing the nuances of neonatal respiratory failure. By systematically analyzing clinical variables and their correlation with respiratory outcomes, it became apparent that an integrative approach that considers both the age of the infant and other key clinical factors can provide a more accurate prediction of disease severity. The resulting N-CRAS, with its straightforward calculation and clear interpretative framework, holds promise as a practical tool for the early identification of high-risk neonates and young infants.

In summary, neonates and young infants’ respiratory pathology presents a complex clinical scenario characterized by high morbidity and significant variability in presentation. The need for effective risk stratification is critical, as it can enable targeted interventions and improve outcomes in this vulnerable population. The development of the N-CRAS represents an important advancement in this regard, offering a simple, age-based tool that integrates key clinical indicators to provide a robust assessment of risk. As the N-CRAS is further validated and refined, it has the potential to become an integral part of neonatal care, guiding both clinical management and future research endeavors. Thus, the present study aimed to develop and perform a preliminary validation of the N-CRAS, a simple bedside tool for the early identification of infants at high risk for severe respiratory outcomes. We hypothesized that a composite score based on routinely available clinical variables, namely respiratory infection, metabolic pathology, symptomatic treatment, mechanical ventilation, and intubation, would correlate with respiratory disease severity and effectively discriminate between risk categories in early infancy.

## 2. Materials and Methods

### 2.1. Study Design and Setting

This prospective observational study was conducted at the Department of Pediatrics of the Emergency County Hospital in Craiova, Romania, over a two-year period (January 2023 to December 2024). The primary aim was to develop and assess the performance of a clinical risk stratification score (N-CRAS) for neonates and young infants presenting with respiratory distress.

All clinical and diagnostic variables were defined according to standard pediatric terminology. A glossary of key operational definitions is clarified in the text, particularly for birth characteristics, feeding types, and diagnostic categories.

### 2.2. Participants and Inclusion Criteria

We included 40 infants aged between 10 and 105 days who were admitted with clinical signs of respiratory compromise, such as tachypnea, nasal flaring, expiratory grunting, and intercostal retractions. The age range encompassed both neonates (defined as ≤28 days of life) and young infants beyond the neonatal period. Although the primary focus of this study was on respiratory conditions occurring during the neonatal window, the inclusion criteria were extended in order to capture delayed or prolonged manifestations of perinatal respiratory pathology.

Importantly, the decision to include patients older than 28 days was also driven by the clinical trajectory of many enrolled cases: a significant proportion of infants required prolonged supportive care, including extended hospital stays and intensive respiratory management. As a result, their age at the time of peak clinical severity or intervention occasionally exceeded the neonatal limit, despite the underlying pathology being perinatal in origin. This broader inclusion strategy enabled a more representative and clinically relevant assessment of early postnatal respiratory morbidity.

Although the term “newborn” typically refers to infants aged 0–28 days, in the context of this study, we used it to designate infants hospitalized with respiratory pathology within the first 105 days of life, reflecting the neonatal profile of patients typically managed in intensive care neonatal units. This broader operational definition allowed the inclusion of clinically relevant cases whose symptom onset or hospitalization occurred shortly after the neonatal period. The inclusion of patients beyond the first 28 days of life and with associated comorbidities was motivated by the clinical reality encountered in the neonatal intensive care unit, where respiratory pathology is often accompanied by metabolic, infectious, or nutritional complications. Therefore, our cohort reflects a pragmatic and integrative model of patient care, aiming to identify early markers of respiratory decompensation even in the presence of multifactorial influences.

No formal exclusion criteria were applied in order to capture the full spectrum of early respiratory illness severity encountered in neonatal and infant patients admitted to our unit.

### 2.3. Variables and Data Collection

Detailed demographic and perinatal information was collected prospectively for all study infants. These variables included age at admission, sex, country of birth, background (urban or rural), and birth order. The ages of the patients ranged from 10 to 105 days, with a mean of 33.15 days and a median of 25 days. For analysis purposes, the cohort was divided into four age groups: <14 days, 15–28 days, 29–42 days, and >43 days. This stratification facilitated a more nuanced exploration of early postnatal respiratory morbidity across developmental stages.

Each patient underwent a comprehensive clinical evaluation upon admission, including documentation of vital respiratory signs and targeted investigations. Clinical variables were collected to reflect both the severity of the respiratory condition and the presence of relevant comorbidities. The following variables were used:

- Respiratory infections: defined by clinical criteria (tachypnea, retractions, and grunting) and supported by laboratory data (e.g., elevated CRP or white blood cell count); microbiological confirmation through viral PCR or bacterial culture was recorded when available;
- Metabolic diseases: included acute imbalances such as hypoglycemia or electrolyte disturbances, as well as diagnosed congenital metabolic disorders identified through neonatal screening or specialist evaluation;
- Symptomatic treatment: referred to supportive, non-etiologic therapy including bronchodilators, antipyretics, or fluid resuscitation administered during inpatient care;
- Mechanical ventilation and intubation: documented when advanced respiratory support was initiated, either non-invasive (NIPPV) or invasive (intubation with mechanical ventilation).

The variable “breathing support” included both low-flow oxygen supplementation and non-invasive positive pressure modalities such as nasal continuous positive airway pressure (CPAP) or non-invasive positive pressure ventilation (NIPPV), as documented in the patient records. The indication for support was based on clinical signs of respiratory compromise, including hypoxia, increased work of breathing, and abnormal respiratory patterns. Although the presence of support was systematically recorded, detailed data on the duration of respiratory assistance were inconsistently documented and therefore were not included in the current analysis. This limitation was noted and will be addressed in future prospective studies.

These five variables were used to construct the Neonatal Clinical Risk Assessment Score (N-CRAS). Each variable contributed one point, yielding a total score ranging from 0 to 5. Based on the cumulative score, patients were stratified into three categories: low risk (0–1 points), moderate risk (2–3 points), and high risk (4–5 points).

All data were anonymized to preserve patient confidentiality and were analyzed using dedicated statistical software to ensure consistency and reproducibility. The selected variables provided both the clinical foundation for developing the N-CRAS and the primary input for subsequent inferential statistical analyses.

All clinical and demographic variables were operationally defined based on institutional protocols and standard neonatal care practices. Specific definitions are provided where applicable in this section.

### 2.4. Definition and Calculation of the N-CRAS

The Neonatal Clinical Risk Assessment Score (N-CRAS) was designed as a composite index to quantify the severity of respiratory pathology in neonates and young infants based on variables available at the time of hospital admission. The score was developed following an exploratory analysis that identified five clinical indicators with significant associations to disease progression and respiratory support needs.

The included variables were as follows:

1. Presence of respiratory infection;
2. Presence of a metabolic disorder;
3. Administration of symptomatic treatment;
4. Requirement for mechanical ventilation;
5. Endotracheal intubation.

Each of these indicators was binary (present or absent) and contributed one point to the final score. Thus, N-CRAS values ranged from 0 (no severity markers) to 5 (all markers present). Based on cumulative scores, patients were stratified into the following clinical risk categories:

- Low risk: 0–1 points;
- Moderate risk: 2–3 points;
- High risk: 4–5 points.

The rationale behind the inclusion of these specific variables was twofold: (1) they are readily available and routinely assessed in early neonatal and infant care, and (2) they demonstrated consistent association with disease complexity across both clinical observations and statistical tests (Chi-square and Spearman correlation). Notably, variables such as mechanical ventilation and intubation served as proxies for high clinical acuity, while symptomatic treatment and metabolic disturbances provided insight into underlying comorbidities and systemic involvement.

Although each component of the score may not independently denote a severe condition, their cumulative presence reflects increasing clinical complexity and a higher likelihood of deterioration. The score is intended as a cumulative severity marker rather than a diagnostic classifier for individual variables.

The simplicity of the scoring system allows for rapid bedside application without requiring advanced diagnostics. Importantly, the N-CRAS was intended not only to reflect the current status of the patient but also to serve as an early triage tool for predicting the potential need for invasive respiratory support.

### 2.5. Statistical Analysis

Descriptive statistics were used to summarize the demographic and clinical characteristics of the study cohort. Continuous variables were expressed as means and standard deviations, along with medians and interquartile ranges where appropriate. Categorical variables were reported as absolute frequencies and percentages.

The Shapiro–Wilk test was applied to assess the normality of continuous variables. Due to the non-normal distribution of key data, non-parametric statistical methods were employed throughout the analysis. Associations between categorical variables were analyzed using either the Pearson Chi-square test or Fisher’s exact test, depending on the distribution of values in contingency tables. Fisher’s test was applied in all situations where expected cell counts were small, ensuring statistical validity in the context of a limited sample size. Spearman’s rank correlation coefficient was used to assess monotonic relationships between ordinal or non-normally distributed variables. A *p*-value of less than 0.05 was considered statistically significant.

Logistic regression was initially explored to assess the predictive value of the N-CRAS; however, due to perfect separation—where all intubated patients had high scores—the model was not statistically estimable in its standard form. Consequently, the discriminative performance of the score was evaluated using receiver operating characteristic (ROC) curve analysis. The area under the ROC curve (AUC) was used to assess predictive accuracy. All statistical analyses were conducted using dedicated software to ensure consistency and reproducibility.

### 2.6. Ethical Approval and Data Protection

The study protocol was approved by the Institutional Ethics Committee of the Emergency County Hospital of Craiova (approval number 15.251/3 April 2025). Written informed consent was obtained from the parents or legal guardians of all the enrolled participants. All data were anonymized prior to analysis and handled in accordance with applicable ethical standards and data protection regulations.

For the purposes of this study, the primary clinical outcome was defined as the need for respiratory support—specifically, mechanical ventilation or intubation—serving as a proxy for disease severity. This outcome was used in the internal validation process of the N-CRAS.

**Figure 1:**
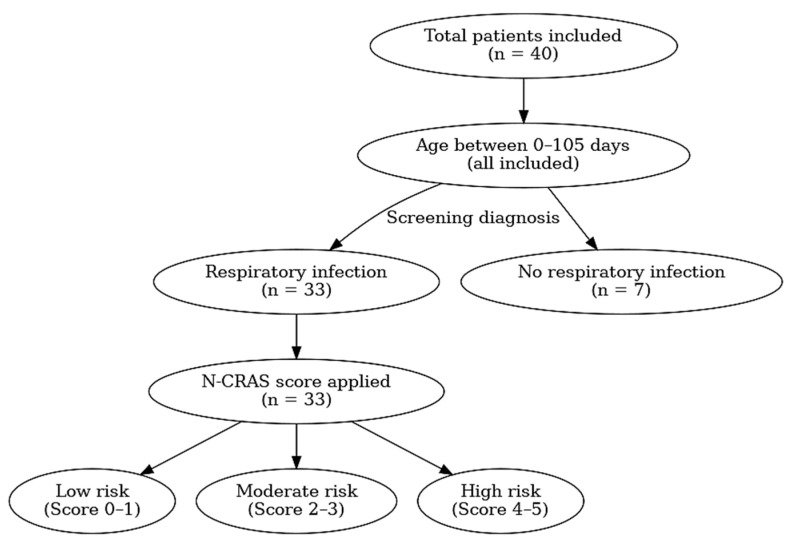
Schematic overview of the study enrollment, diagnostic grouping, and N-CRAS application.

For transparency, a schematic overview of the study enrollment and patient classification process is presented in Figure 1. This diagram outlines the inclusion flow, diagnostic grouping, and application of the N-CRAS scoring system.

## 3. Results

### 3.1. Study Population

The sample analyzed included 40 patients. The mean age at admission was 33.15 ± 24.77 days. The minimum and maximum ages observed were 10 and 105 days, respectively. A percentile analysis showed that 25% of the patients were younger than 19.5 days, the median age (50th percentile) was 25 days, and 75% of the patients were younger than 36.75 days.

We used the interquartile range (IQR) method to identify extreme values. We determined lower and upper thresholds to identify patients with unusually high or low ages. This revealed four extreme values, corresponding to the ages of 84, 104, and 105 days, which exceeded the range defined by the IQR.

The Shapiro–Wilk normality test was applied to assess the variable distribution. The test results showed a *p*-value = 3.49 × 10^−7^, suggesting that the age distribution at admission does not follow a normal distribution (*p* < 0.05). This means that the data is not normally distributed, and therefore, non-parametric statistical tests should be used in further analysis.

Age was coded into interval classes for further data analysis. Thus, according to the children’s ages, four classes were obtained: class 1—newborns under 14 days of age; class 2—newborns aged 15–28 days; class 3—patients aged 29–42 days; and class 4—patients aged over 43 days.

The age distribution of patients revealed a balanced distribution across the four distinct categories. Most cases fell into the third age group (37.5% of patients), followed closely by the second (32.5%) and first (20%). Only 10% of the patients were included in the fourth age class, which corresponds to the oldest ages observed, maintaining the balance in the data.

Thus, patients in the first age group (*n* = 8, 20% of all patients) had a mean age of 11.88 ± 1.81 days and a median of 11 days. In the second age group (*n* = 13, 32.5% of all patients), the mean was 21.38 ± 2.90 days, and the median was 21 days, indicating a slight increase from the first group. Class three (*n* = 15, 37.5% of all patients) had a mean age of 37 ± 4.08 days and a median of 36 days, reflecting a relatively homogeneous distribution of patients. In contrast, the fourth class (*n* = 4, 10% of all patients) showed increased variability, with a mean age of 99.5 ± 10.34 days and a median of 104.5 days, confirming the presence of extreme values in this category.

The analysis of the distribution of patients according to gender and age group showed a male preponderance (62.5% of cases) compared to female (37.5%), as seen in Table 1. Regarding the age distribution, 7.5% of girls and 12.5% of boys were hospitalized within 14 days. Between 15 and 28 days, the proportion of girls and boys was 7.5% and 25%, respectively. The distribution was relatively even between 28 and 42 days, with 20% of girls and 17.5% of boys. Girls were less represented in the category over 43 days (2.5%) than boys (7.5%).

A Chi-square test was used to assess the association between gender and age class. The Pearson Chi-square test result showed a value of 3.025 (*p* = 0.388), indicating that there is no statistically significant association between the two variables. The results indicate no significant relationship between gender and age, with neither having a notable influence on the other.

The distribution of patients according to country of birth showed a predominance of newborns from the country (95%) compared to those born abroad (5%) in terms of distribution by age group. Patients born in the country were more frequently hospitalized between 15 and 28 days (30%) and between 28 and 42 days (37.5%). In contrast, among patients born internationally, only two cases were recorded. One participant was in the 15–28 days group (2.5%), and another was in the group with over 43 days (2.5%).

A Chi-square test was conducted to evaluate the relationship between country of birth and age class. The Pearson Chi-square test result showed a value of 4.777 (*p* = 0.189), suggesting that there is no statistically significant association between the two variables. Also, the Spearman correlation showed a weak and insignificant relationship between country of birth and age (coefficient of 0.131, *p* = 0.422).

The analysis of the distribution of patients according to origin and age group showed a higher proportion of rural patients (62.5%) compared to urban patients (37.5%). Regarding age distribution, rural patients were more frequently hospitalized between 15 and 28 days (25%) and between 28 and 42 days (22.5%). In contrast, urban patients had a more balanced distribution, with a similar representation in all age groups.

A Chi-square test was used to assess the association between background and age class. The Pearson Chi-square test result showed a value of 3.594 (*p* = 0.309), suggesting that there is no statistically significant association between the two variables. The Spearman correlation also revealed a weak and insignificant relationship between background and age (coefficient of 0.191, *p* = 0.239).

The distribution of patients by birth order showed a variable representation within each age group. The second (30%) and third (22.5%) orders were most frequently hospitalized, followed by the first (17.5%). The higher orders (4, 5, and 6) were less represented, with percentages below 10%.

A statistical analysis did not reveal a significant association between child order and age class distribution. The Chi-square test showed a value of 18.018 (*p* = 0.454), suggesting no significant relationship between these variables. Spearman’s correlation also showed a weak association (coefficient −0.054, *p* = 0.739), indicating that child order does not significantly influence the age group distribution.

### 3.2. Clinical Interventions and Observations

The distribution analysis based on environment of origin and birth weight classification showed that the majority of patients were from rural areas (62.5%), with normal-weight neonates being more frequent in both rural (37.5%) and urban (25.0%) settings. First-degree preterm infants were more commonly observed in rural (15.0%) than urban (5.0%) environments. However, Fisher’s exact test revealed no statistically significant association between the environment of origin and birth weight category (*p* = 0.678).

The data showed that the birth order and weight classification of patients suggested that the first two children in a family had a higher likelihood of being classified as normal weight. In contrast, children with higher birth orders (3, 4, 5, and 6) had a higher frequency of prematurity. The Chi-square test showed a significant association between child order and weight classification (*p* < 0.0001), suggesting that birth order may influence birth weight.

Pregnancy monitoring was associated with birth weight, revealing that most normal-weight patients (45%) were from pregnancies with adequate prenatal care. In contrast, grade 1 and 2 preterm infants were more frequently recorded among those with inadequate or incomplete monitoring. Fisher’s exact test confirmed a statistically significant association between the type of pregnancy monitoring and birth weight (*p* = 0.018), highlighting the potential impact of antenatal care quality on neonatal outcomes.

Among the study cohort, natural births were more frequently associated with normal-weight newborns (35%) than cesarean deliveries (27.5%). Interestingly, preterm infants—particularly those classified as grade 1 and 2—were also more commonly delivered via natural birth than cesarean section. The association between birth type and neonatal weight category was statistically significant, as confirmed by the Chi-square test (*p* < 0.0001), suggesting that mode of delivery may reflect underlying perinatal risk factors.

The distribution of patients by gestational age and weight classification showed that term deliveries were most commonly associated with normal-weight newborns (20.5%). Conversely, preterm births were more frequently linked to grade 1 (10.3%) and grade 2 (5.1%) premature infants. Deliveries occurring between 34 and 37 weeks presented a similar distribution pattern to term births but with a lower frequency of normal-weight neonates (10.3%). The Chi-square test did not reveal a statistically significant association between gestational age at delivery and neonatal weight classification (*p* = 0.240), and Spearman’s correlation showed a weak inverse relationship (r = −0.209, *p* = 0.202), suggesting no clear trend between the two variables.

The analysis of the distribution of patients according to their birth length and weight classification indicated that most normal-weight newborns had a length between 48 and 52 cm (35%). Grade 1 and 2 preterm newborns were more frequently recorded with a length below 48 cm (10% and 2.5%, respectively). In 42.5% of cases, information on birth length was unavailable. The Chi-square test showed a significant association between newborn waist and weight classification (*p* = 0.012), and the Spearman correlation showed a moderate association between these variables (coefficient 0.419, *p* = 0.007). This result suggests that birth weight may be a relevant indicator of neonatal weight. The analysis of the relationship between APGAR score and weight classification showed that most normal-weight newborns had higher APGAR scores (42.5% in the APGAR good score category). In contrast, grade 1 and 2 preterm infants were more frequent in the good APGAR score group (17.5% and 2.5%). A significant percentage of patients for whom APGAR score information was unavailable were distributed among all weight categories. The Chi-square test showed a significant association between APGAR score and weight classification (*p* = 0.018), and the Spearman correlation showed a moderate association between these variables (coefficient 0.422, *p* = 0.007). These results suggest that the APGAR score may be a relevant indicator of neonatal clinical status and birth weight.

The analysis of the relationship between the presence of neonatal jaundice and weight classification showed that 42.5% of patients had jaundice, and it was more common among normal-weight infants (27.5%) and grade 1 and 2 preterm infants (10% and 2.5%). In contrast, in the group without jaundice, most patients were of normal weight (35%), and the frequency of preterm infants was lower. The Chi-square test did not reveal a significant association between jaundice and weight classification (*p* = 0.158). However, Spearman’s correlation indicated a moderately positive association between these variables (coefficient 0.337, *p* = 0.033), suggesting that jaundice may be more common in preterm and lower birth weight infants.

An analysis of the relationship between type of feeding and weight classification showed that most normal-weight infants were naturally breastfed (27.5%). Mixed feeding (breast milk and formula) was recorded in 7.5% of grade 1 and 2 preterm infants, while exclusively formula-fed newborns accounted for 7.5% of the cohort. Notably, data on diet type were missing in 30% of cases. Although these proportions suggest some variability across feeding categories, the association between diet type and weight classification did not reach statistical significance. Fisher’s exact test yielded a *p*-value of 0.643. Similarly, Spearman’s correlation indicated a weak and non-significant relationship between the two variables (coefficient 0.186, *p* = 0.251). These findings suggest that while feeding practices may vary with gestational maturity, diet type does not appear to be a strong predictor of birth weight in this sample.

The analysis of the relationship between the length of hospitalization and weight classification revealed that 42.5% of cases lacked data on hospitalization duration. Among normal-weight newborns, most were hospitalized for 1 to 7 days. Grade 1 and 2 preterm infants tended to experience longer hospital stays, although the distribution was uneven across different duration intervals. Fisher’s exact test did not reveal a statistically significant association (*p* = 0.989). Spearman’s correlation analysis also confirmed the absence of a significant relationship between these variables (coefficient 0.006, *p* = 0.970). These findings suggest that the duration of hospitalization is not directly influenced by neonatal weight status, but it may depend on other underlying clinical or logistical factors. (Table 2).

The analysis of the relationship between antenatal monitoring and the type of delivery revealed a statistically significant association (Chi-square test, *p* = 0.004), as shown in Table 3. Pregnant women with adequate prenatal monitoring had the highest frequency of natural births (32.5%) and cesarean deliveries (22.5%), while those with no or incomplete monitoring were less likely to undergo either type of birth (natural births: 10.0% and 2.5%, respectively; cesarean births: 5.0% and 2.5%, respectively). The Spearman correlation coefficient confirmed a moderate positive relationship (r = 0.485, *p* = 0.001), suggesting that a higher level of antenatal care may be associated with the likelihood and type of delivery.

Concerning the association between background and pregnancy monitoring, although there was a tendency for urban pregnant women to be more frequently discharged (22.5% compared to 32.5% in rural areas), statistical analysis did not identify a significant association (*p* = 0.188). Spearman’s correlation was weak and insignificant (r = 0.193, *p* = 0.232).

In order to contextualize the clinical status of the patients included in this study, several relevant characteristics were analyzed according to the age at admission or onset of symptoms (<14 days, 15–28 days, 28–42 days, and >43 days), as shown in Table 4.

Neonatal jaundice was present in 15 patients (37.5%), relatively evenly distributed over the age ranges. There was no statistically significant association between the presence of jaundice and age (*p* = 0.475), and the Spearman correlation was weakly negative and insignificant (r = −0.150, *p* = 0.357).

In terms of incubator requirements, eight patients (20%) required incubator admission, predominantly in the first 28 days. There were no significant differences between groups (*p* = 0.162), and the correlation was very weak (r = 0.037, *p* = 0.821).

For phototherapy, applied in 12.8% of the cases, there was a trend toward an association with younger age, but without statistical significance (*p* = 0.057). Spearman’s correlation was weakly negative (r = −0.164, *p* = 0.318).

In contrast, mechanical ventilation and tracheal intubation were significantly associated with patient age. Of those who required ventilation (10%), the majority were either less than 14 days (2.5%) or more than 43 days (7.5%), with no cases in the intermediate groups. The same distribution was observed for intubated patients. Fisher’s exact test showed a statistically significant association (*p* = 0.008 for both variables), although Spearman correlations were moderate and marginally insignificant (r = 0.288, *p* = 0.071).

Some significant differences were identified in analyzing the relationship between the age group at admission and various neonatal pathologies. Respiratory infections were the most frequent, occurring in 82.5% of patients, with an unequal distribution between groups: predominant in the 28–42 days group (42.4%) and followed by the <14 days group (24.2%). The Chi-square test showed a significant association between the presence of respiratory infections and age group (*p* = 0.011), suggesting that the patient’s age at the onset of symptoms or admission may influence the prevalence of this diagnosis. However, the Spearman correlation coefficient did not show a statistically significant relationship, indicating that the observed association is not linear.

Respiratory complications were present in all patients and were thus a constant variable. For this reason, a comparative statistical analysis between groups could not be performed. Septal defects were identified in 15% of patients, with no significant differences between age groups (Fisher’s exact test *p* = 0.355). Other cardiac disorders were also rare, being identified in only one case (2.5%), which limits the possibility of relevant statistical interpretation.

Metabolic diseases were reported in 10% of cases, being present exclusively in patients aged 15–28 days. The analysis showed a significant association between this pathology and age group (Fisher’s exact test, *p* = 0.026), which may reflect the usual clinical onset period for some congenital metabolic disorders. Spearman correlations were, however, insignificant, indicating that the distribution does not follow a progressive pattern.

Hematologic diseases were reported in 20% of the cases, with a relatively uniform distribution among age groups and without significant association (Chi-square test, *p* = 0.439). In contrast, nutritional diseases were present in all patients in the analyzed group, constituting a constant variable that did not allow statistical testing of associations with age (Table 5).

### 3.3. Therapeutic Approach According to Diagnoses

Antibiotic administration was almost uniform across all age groups, with no statistically significant differences (Chi-square test, *p* = 0.821). Antibiotics were used in 100% of patients under 14 days and over 43 days age groups, 96.0% in the 15–28 days group, and 93.3% in the 28–42 days group. Spearman correlations showed no significant association (r = −0.031, *p* = 0.848).

Antiviral/antifungal treatment was rarely used, especially in the age groups 15–28 days, 28–42 days, and over 43 days, where one patient (2.5%) in each group received this type of treatment. It was not given at all in the under 14 days age group. The Chi-square test was not significant (Fisher’s exact test, *p* = 0.488), and Spearman’s correlation was weak (r = 0.182, *p* = 0.262).

The administration of corticosteroids and anti-inflammatory drugs tended to be associated with age (Fisher’s exact test, *p* = 0.080). These were administered to all patients in the under 14 days group, 61.5% in the 15–28 days group, 46.7% in the 28–42 days group, and 75.0% in the over 43 days group. Spearman’s correlation was moderately negative (r = −0.279, *p* = 0.081).

Symptomatic treatment had a statistically significant association with age (Fisher’s exact test, *p* = 0.047). It was absent in the under 14 days group, administered in 23.1% of cases in the 15–28 day group, 33.3% in the 28–42 day group, and 75.0% in the over 43 day group. Spearman’s correlation was positive and significant (r = 0.408, *p* = 0.009).

Respiratory support was used in a higher proportion in the 15–28 days (69.2%) and 28–42 days (60.0%) age groups, compared with 87.5% in the under 14 days group and 100% in the over 43 days group. However, these differences were not statistically significant (Chi-square test, *p* = 0.300), and the Spearman correlation was weakly negative (r = −0.066, *p* = 0.684).

Gastrointestinal treatments were more frequently used in the 15–28 days (23.1%) and 28–42 days (26.7%) groups, being absent in the under 14 days group and present in only one patient (25.0%) in the over 43 days group. Fisher’s exact test showed no significant association (*p* = 0.465), and Spearman’s correlation was insignificant (r = 0.202, *p* = 0.211).

Sedatives/anesthetics were administered in only one case, in the age group over 43 days, and in the other groups, not at all. Although Fisher’s exact test was significant (*p* = 0.026), the small number of cases limits the interpretative value. Spearman’s correlation was not significant (r = 0.263, *p* = 0.102).

Vitamins and supplements were administered only in the 28–42 days age group (2 cases, 13.3%) and were not used in the other groups. This distribution was not statistically significant (Fisher’s exact test, *p* = 0.320), and the Spearman correlation was insignificant (r = 0.178, *p* = 0.273).

Local treatment was more frequent in the 28–42 days (46.7%) and 15–28 days (23.1%) groups, being present in 25.0% of cases under 14 days and 25.0% in the group over 43 days. The distribution was not statistically significant (Fisher’s exact test, *p* = 0.531), and the Spearman correlation was weak (r = 0.141, *p* = 0.386) (Table 6).

### 3.4. N-CRAS—Neonatal Clinical Risk Assessment Score

Based on the results obtained in the primary analysis, we proposed a new clinical score, called the N-CRAS (Neonatal Clinical Risk Assessment Score), developed as a clinical stratification tool according to the severity of respiratory conditions in the newborn. The score was designed following an exploratory statistical analysis, which identified variables significantly associated with age and clinical case complexity: respiratory infections, metabolic diseases, symptomatic treatment, mechanical ventilation, and intubation. These items were selected because of their clinical relevance and their predictive value demonstrated in inferential analysis (Chi-square and Spearman correlation) (Table 7).

The N-CRAS is calculated by assigning one point for the presence of each of the five variables mentioned, resulting in a total score between 0 and 5 points. Based on the value obtained, patients are placed in one of the following risk categories: low, moderate, and high, and the distribution of scores is as follows:

- 0–1 points → low risk.
- 2–3 points → moderate risk.
- 4–5 points → high risk.

The application of the N-CRAS is straightforward. Based on data available from the first hours of hospitalization, it does not require further investigations. The interpretation of the score provides a rapid tool for guiding therapeutic decisions, prioritizing resources, and monitoring patient evolution over time. Thus, the score can be used as a clinical triage and an indicator of severity in future research.

The distribution of the N-CRAS according to age classes showed a progressive transition from low values in the <14 days group to higher scores in the 28–42 days and >43 days group. The Chi-square test applied for the association between score and age class was significant (*p* < 0.05), and the Spearman correlation showed a positive trend, suggesting that the score increases with patient age. This score distribution is shown in Table 8.

Importantly, the N-CRAS demonstrated excellent predictive power for intubation requirements. In the inferential analysis, logistic regression could not be applied classically because of a perfect separation between high scores and the presence of intubation, a phenomenon that clearly emphasizes the score’s ability to discriminate severe cases. This perfect separation indicates that all intubated patients had high scores with no overlap with non-intubated patients, supporting the score’s practical and clinical value.

**Figure 2:**
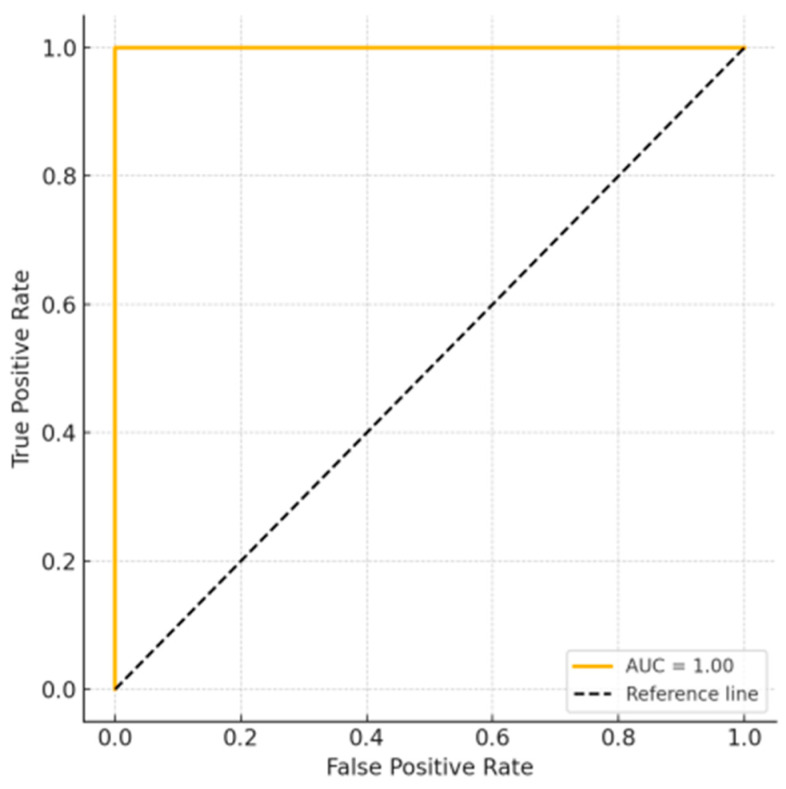
ROC curve of the N-CRAS for predicting intubation requirements in neonates and young infants.

The analysis was completed by plotting the ROC curve for the prediction of intubation as a function of the N-CRAS. The result showed an AUC (area under the curve) of 1.00, reflecting a perfect predictive ability in the sample studied. The ROC curve is shown in Figure 2, appended to the article, and highlights the robustness of the score in discriminating cases with severe evolution and reinforces the recommendation of its use as a clinical tool for early triage. While the AUC of 1.00 suggests excellent model performance, external validation is required to confirm its robustness.

While the N-CRAS demonstrated a strong alignment with observed clinical severity—especially in high-risk patients requiring intubation or mechanical ventilation—it must be emphasized that this study represents a preliminary validation. No external comparator or gold standard score was included, and no correlation with long-term outcomes could be established. Therefore, the score should be interpreted as an initial risk stratification tool, pending validation in a larger, prospective cohort.

## 4. Discussion

The statistical analysis of the variables studied in this article revealed several relevant aspects for understanding the factors involved in the clinical course of newborns. The data collected allowed a detailed investigation of demographic characteristics, neonatal history, and their impact on perinatal health status.

Although only 15 patients in the cohort met the strict definition of neonates (≤28 days of age), the decision to include young infants up to 105 days was based on clinical experience indicating that respiratory complications may emerge or evolve subacutely beyond the neonatal period. This broader inclusion allowed for a more comprehensive evaluation of early postnatal respiratory risk.

Statistical analysis of age at admission and age classes suggests an asymmetric distribution with extreme values. The age distribution is not normal, implying the need for non-parametric analysis methods in further studies. Classification by age groups allows a better understanding of patient characteristics and may facilitate the interpretation of risk factors associated with acute respiratory failure in neonates and young infants. Furthermore, the analysis of the relationship between country of birth, background, child order, and age indicated no significant correlations, implying that age distribution at admission does not depend on these demographic factors. The age distribution at admission was asymmetric, with a significant presence of extreme values, indicating the need for a detailed analysis of the causes of late or prolonged hospitalizations. Classification by age groups allowed the identification of significant differences in the duration of hospitalization and the clinical characteristics of patients.

Several demographic and perinatal factors were found to significantly impact birth weight. A clear association was observed between birth order and prematurity, suggesting that birth order may influence the risk of prematurity. The type of pregnancy monitoring was also found to be a relevant predictor of birth weight, emphasizing the importance of regular antenatal care in preventing prematurity and neonatal complications.

A significant link was found between birth type and weight, with more preterm births in natural deliveries than in cesarean sections. This result may suggest that obstetric interventions are important in reducing the risks associated with prematurity.

Newborn height has been identified as a significant predictor of neonatal weight, confirming this parameter’s usefulness in the initial weight status assessment. The APGAR score also demonstrated a moderate association with weight classification, indicating that clinical status at birth may indirectly reflect the risks associated with prematurity and low birth weight.

The analysis also revealed that neonatal jaundice was more common in preterm and low-birth-weight infants, emphasizing the need for a rigorous monitoring protocol for these patients. On the other hand, the type of neonatal feeding did not have a statistically significant association with birth weight. However, it may influence the neonate’s nutritional status and overall outcome over time. Hospitalization length did not correlate directly with birth weight, indicating that clinical factors and specific care needs affect this measure.

The analysis revealed a significant association between antenatal monitoring and type of delivery, with a higher proportion of natural and cesarean births among pregnant women. This result supports the hypothesis that appropriate antenatal care may influence obstetric management, facilitating more informed medical decisions.

In terms of background, there was no significant association with the type of pregnancy monitoring, although there was a trend toward more frequent dispensing in urban areas. This finding is limited by the small sample size and does not influence the study’s main conclusions.

Data on perinatal clinical history indicates a significant association between the patient’s age and the need for mechanical ventilation or intubation, with these being more frequent at the extremes of the age ranges analyzed (<14 days and >43 days). These results may suggest a higher risk of severe respiratory complications in these groups or delays in presenting more advanced cases to the hospital. In contrast, jaundice, phototherapy, and incubator requirement did not show significant associations with age, which may reflect a relatively even distribution of these conditions over the extended neonatal period.

The only statistically significant associations observed were between age group and respiratory infections (*p* = 0.011) and metabolic diseases (*p* = 0.026), respectively. Respiratory infections were predominant in infants aged 28–42 days, suggesting a possible window of immunologic vulnerability or late diagnosis. For metabolic diseases, the exclusive presence in the 15–28-day group might reflect the usual age of clinical onset for some previously undiagnosed congenital disorders. The remaining disorders did not show significant age-dependent variations.

A detailed analysis showed that the patient’s age at the time of hospitalization significantly influences some clinical aspects and the distribution of certain treatments. Although demographic parameters such as background or antenatal follow-up were not significantly correlated with most pathologies, a significant association was observed between the type of delivery and pregnancy discharge, suggesting a positive impact of antenatal follow-up on mode of delivery.

Age is an important factor in the prevalence of respiratory infections, with increased values, especially in the age groups below 14 days and above 28 days. This could reflect the increased vulnerability of newborns in these age ranges. Metabolic diseases appeared only in the 15–28 day age group, suggesting a critical period for congenital disorders.

Symptomatic treatment was significantly associated with age, increasing in frequency in higher age groups. Although frequently used, corticosteroids and anti-inflammatory drugs had a less clear association with age, while antibiotics were almost universally administered, irrespective of group. Other therapies, such as antivirals, sedatives, or supplements, were used occasionally and without clear patterns.

The presence of specific pathologies also influenced the distribution of treatments. For example, patients with respiratory infections frequently received corticosteroids and respiratory support, whereas patients with septal defects more often received symptomatic treatments. Some diseases were constant in the whole group (e.g., respiratory complications and nutritional diseases), which limited the statistical analysis but emphasized a standard clinical profile in the study population.

Overall, the results indicate that age plays a relevant role in the clinical and therapeutic profile of the newborn. Although not all the differences identified were statistically significant, the observed trends may contribute to optimal decision making and better individualization.

In this observational study, the statistical analysis revealed significant associations between neonatal age and certain clinical characteristics and the development of a clinical tool derived directly from the data obtained—the N-CRAS. The selection of the variables composing the score was based solely on their statistical and clinical relevance, reflecting a data-driven approach with immediate applicability in patient triage. The N-CRAS demonstrated excellent predictive ability for clinical severity (intubation), which gives it the potential to be integrated as a risk marker in current neonatal practice. Internal validation by the ROC curve and perfect separation identified by logistic regression reinforce the robustness of this tool in the study group.

Although the N-CRAS provided promising results, this study also has some limitations. First, the score was validated on a single group of patients, which may limit the generalizability of the results. Second, the components of the score may be influenced by local particularities of clinical practice or the way data are recorded. External validation in larger and heterogeneous populations is therefore necessary.

The N-CRAS can be applied not only as a clinical tool for early triage but also as a risk stratification method in prospective studies to assess the effectiveness of therapeutic interventions. By being integrated into clinical assessment protocols, the score could contribute to a more efficient allocation of resources and personalization of interventions.

The score’s simplicity and the fact that it utilizes only available data at admission make it highly feasible in various clinical settings, including neonatal care units with limited resources. The lack of need for further investigations makes it practical and reproducible, favoring its widespread adoption.

The newly introduced Neonatal Clinical Risk Assessment Score provides a comprehensive risk stratification framework, and its performance can be contextualized by comparing it to established neonatal scoring systems. Traditional respiratory scores such as the Silverman–Anderson and Downes scores have long served as bedside tools for assessing the severity of respiratory distress based on observable clinical signs. Although these systems are appreciated for their simplicity and ease of use, they are limited by their focus solely on physical examination findings and do not incorporate additional risk factors such as gestational maturity or perinatal history. Broader indices, like SNAP-II and CRIB-II, have been developed to predict overall illness severity and mortality risk. However, these indices are general in nature and often lack the specificity required for addressing respiratory failure. The N-CRAS bridges this gap by combining respiratory-specific indicators with essential demographic and clinical variables, thereby providing a tailored risk assessment for neonates and young infants with respiratory distress [27,28,29,30].

One of the critical strengths of the N-CRAS is its emphasis on age-related risk stratification. It is well known that the maturity of the neonatal respiratory system plays a pivotal role in determining outcomes. Premature infants, with their underdeveloped lungs and surfactant deficiency, are at a much higher risk for conditions like respiratory distress syndrome, which can lead to prolonged mechanical ventilation and chronic lung disease. Conversely, full-term infants, though generally at lower risk for such complications, may still experience significant respiratory challenges due to infections or aspiration events. By stratifying risk based on distinct age groups, the N-CRAS recognizes that the underlying pathophysiology and potential for deterioration differ markedly between very preterm and near-term or term neonates and young infants. This age-sensitive approach allows clinicians to anticipate the likely trajectory of the disease and to tailor interventions accordingly, ensuring that the most vulnerable infants are identified early and managed with appropriate urgency. The clinical implications of integrating the N-CRAS into routine practice are substantial. Early risk stratification is paramount in the neonatal intensive care unit, where timely decision making can dramatically alter outcomes. In many cases, the decision to initiate advanced respiratory support or to transfer a neonate to a higher-level care facility is made based on a combination of clinical judgment and limited objective measures. The N-CRAS offers a more systematic method for evaluating risk, thereby potentially reducing reliance on subjective assessment alone. By providing an objective, quantifiable measure of risk early in the course of illness, the score can help guide clinical decisions such as the early initiation of interventions, the allocation of resources, and even the design of personalized care plans for individual infants [31,32,33].

In recent years, the field of neonatology has witnessed a growing trend toward personalized medicine. There is an increasing recognition that one-size-fits-all approaches are often inadequate for the complex and heterogeneous population of neonates and young infants with respiratory distress. Risk scoring systems that incorporate key predictors of outcome have the potential to transform clinical practice by enabling targeted, individualized interventions. The N-CRAS, by incorporating variables such as the presence of respiratory infections, metabolic disturbances, and the need for supportive measures like mechanical ventilation and intubation, exemplifies this shift toward personalized care. It not only assesses the immediate clinical status of the neonate but also offers predictive insight into the likely progression of the disease, thereby supporting more proactive management strategies. Despite its promising performance, the N-CRAS is not without limitations. Its development was based on data from a single center, which may limit the generalizability of the findings. Neonatal populations can vary widely across different settings due to differences in patient demographics, clinical practices, and available resources. External validation in diverse clinical environments is necessary to confirm the score’s utility and to adjust it if required. Furthermore, while the score relies on readily available clinical parameters, some of these variables, such as the assessment of respiratory distress, may be subject to inter-observer variability. Although efforts were made to standardize assessments, inherent subjectivity in clinical evaluations remains a challenge. Future enhancements might incorporate more objective measures, such as continuous monitoring data, to further improve the score’s reliability [34,35,36,37,38,39].

Another consideration is the outcome focus of the N-CRAS. In our study, the score was primarily validated against short-term respiratory outcomes, such as the need for intubation. While this is clinically valuable, it remains to be seen how well N-CRAS predicts longer-term outcomes like the development of chronic lung disease or neurodevelopmental impairment. Continued research is needed to explore the relationship between early risk stratification and longer-term clinical trajectories. Such studies could help refine the score further and establish its role in not only guiding immediate care but also in planning long-term follow-up and intervention strategies.

The potential impact of the N-CRAS extends beyond its predictive capabilities. By providing a simple yet robust tool for risk stratification, it may facilitate more efficient resource allocation in the neonatal intensive care unit. For instance, infants identified as high risk could be prioritized for interventions such as early surfactant therapy or more intensive monitoring, while those at lower risk might be managed with less aggressive approaches. This targeted use of resources is particularly important in settings where NICU capacity is limited and the cost of care is high. Moreover, the development of the N-CRAS contributes to the broader understanding of neonatal respiratory pathology. It reinforces the idea that the interplay between age, respiratory function, and systemic factors is complex and warrants a nuanced approach to risk assessment. By integrating key clinical predictors into a single, composite score, N-CRAS encapsulates the multidimensional nature of neonatal respiratory distress, paving the way for more refined and individualized treatment protocols. As clinical practice evolves and more data become available, there is the potential to further refine the score, perhaps incorporating additional biomarkers or advanced imaging findings to enhance its predictive power.

This study presents several additional limitations that should be acknowledged. First, the sample size was relatively small (*n* = 40), which may limit the statistical power and generalizability of the findings. The perfect discrimination observed in the ROC analysis (AUC = 1.00) could reflect overfitting due to the limited dataset and should be interpreted with caution. Second, the N-CRAS was developed and validated using data from a single center, which may introduce center-specific biases related to clinical practices or patient population characteristics. Third, some clinical variables, such as the decision to initiate mechanical ventilation or symptomatic treatment, may be subject to inter-observer variability, potentially affecting score reliability. Lastly, no external validation or comparison with existing neonatal risk scores was conducted in this preliminary phase. Further multicentric studies with larger and more diverse populations are needed to validate the score’s performance and applicability in broader clinical settings.

That being said, the number of patients who required intubation or mechanical ventilation in our cohort was limited, which inherently restricts the statistical power of the analysis. This constraint may have contributed to the phenomenon of perfect separation observed in the logistic regression model. Although the ROC analysis yielded an AUC of 1.00, which indicates an excellent discriminatory ability, such a result must be interpreted with caution. It is plausible that this finding reflects overfitting due to the small sample size rather than robust predictive accuracy. Taken together, these aspects highlight the preliminary nature of our findings and reinforce the need for external validation in larger, more heterogeneous neonatal populations.

While the N-CRAS demonstrated a promising performance in identifying infants at increased risk of requiring intensive respiratory support, we recognize that this initial validation is limited by the sample size and the absence of a gold standard comparator. As such, the score should be regarded as a preliminary tool, serving as a conceptual and methodological foundation for future studies. Further external validation across larger and more heterogeneous neonatal populations is essential to confirm its predictive accuracy and clinical applicability.

## 5. Conclusions

The study showed relevant associations between neonatal age and the severity of some clinical conditions, particularly respiratory pathology. Age at admission was found to be a factor associated with the need for advanced support, such as mechanical ventilation and intubation. In conclusion, our study revealed several significant correlations between perinatal factors and newborn clinical outcomes. These findings may contribute to improving neonatal prevention and management strategies, providing a basis for optimizing perinatal care and reducing complications associated with prematurity and low birth weight.

The N-CRAS offers a simple, rapid, and clinically relevant tool to stratify respiratory risk in infants presenting with respiratory symptoms. With further validation, it has the potential to improve early clinical decision making and optimize neonatal respiratory care.

## Figures and Tables

**Table 1:** Distribution of demographic data across neonatal and early-infant age groups.

Parameter	Variable	Age (Days)				*p*
		Under 14 Days	15–28 Days	28–42 Days	Over 43 Days	
Child’s gender	F	3 (7.5%)	3 (7.5%)	8 (20.0%)	1 (2.5%)	0.388
	M	5 (12.5%)	10 (25.0%)	7 (17.5%)	3 (7.5%)	
Country of birth	Internal	8 (20.0%)	12 (30.0%)	15 (37.5%)	3 (7.5%)	0.189
	International	0 (0.0%)	1 (2.5%)	0 (0.0%)	1 (2.5%)	
Background	Rural	5 (12.5%)	10 (25.0%)	9 (22.5%)	1 (2.5%)	0.309
	Urban	3 (7.5%)	3 (7.5%)	6 (15.0%)	3 (7.5%)	
Newborn’s order	Unknown	0 (0.0%)	2 (5.0%)	3 (7.5%)	0 (0.0%)	0.454
	1	2 (5.0%)	0 (0.0%)	5 (12.5%)	0 (0.0%)	
	2	3 (7.5%)	4 (10.0%)	4 (10.0%)	1 (2.5%)	
	3	3 (7.5%)	3 (7.5%)	2 (5.0%)	1 (2.5%)	
	4	0 (0.0%)	1 (2.5%)	1 (2.5%)	1 (2.5%)	
	5	0 (0.0%)	2 (5.0%)	0 (0.0%)	1 (2.5%)	
	6	0 (0.0%)	1 (2.5%)	0 (0.0%)	0 (0.0%)	

Chi-square test.

**Table 2:** Distribution of patient history by birth weight.

Parameter	Variable	Weight of Patients					*p*
		No Information	Low Birth Weight	Moderate Preterm	Late Preterm	Extremely Low Birth Weight	
Area of residence	Rural	4 (10.0%)	15 (37.5%)	6 (15.0%)	0 (0.0%)	0 (0.0%)	0.678 *
	Urban	1 (2.5%)	10 (25.0%)	2 (5.0%)	1 (2.5%)	1 (2.5%)	
Child’s order	Unknown	5 (12.5%)	0 (0.0%)	0 (0.0%)	0 (0.0%)	0 (0.0%)	<0.0001 **
	1	0 (0.0%)	5 (12.5%)	2 (5.0%)	0 (0.0%)	0 (0.0%)	
	2	0 (0.0%)	9 (22.5%)	2 (5.0%)	0 (0.0%)	2 (5.0%)	
	3	0 (0.0%)	8 (20.0%)	2 (5.0%)	0 (0.0%)	0 (0.0%)	
	4	0 (0.0%)	2 (5.0%)	2 (5.0%)	0 (0.0%)	0 (0.0%)	
	5	0 (0.0%)	2 (5.0%)	2 (5.0%)	2 (5.0%)	0 (0.0%)	
	6	0 (0.0%)	0 (0.0%)	2 (5.0%)	0 (0.0%)	0 (0.0%)	
Monitoring task	No information	5 (12.5%)	2 (5.0%)	3 (7.5%)	0 (0.0%)	0 (0.0%)	0.018 *
	Adequate prenatal monitoring	0 (0.0%)	18 (45.0%)	3 (7.5%)	0 (0.0%)	1 (2.5%)	
	Lack of prenatal monitoring	0 (0.0%)	3 (7.5%)	2 (5.0%)	1 (2.5%)	0 (0.0%)	
	Incomplete prenatal monitoring	0 (0.0%)	2 (5.0%)	0 (0.0%)	0 (0.0%)	0 (0.0%)	
Type of birth process	No information	5 (12.5%)	0 (0.0%)	0 (0.0%)	0 (0.0%)	0 (0.0%)	<0.0001 **
	Natural birth	0 (0.0%)	14 (35.0%)	7 (17.5%)	1 (2.5%)	1 (2.5%)	
	Cesarean birth	0 (0.0%)	11 (27.5%)	1 (2.5%)	0 (0.0%)	0 (0.0%)	
Type of birth (by gestational age)	Preterm birth	0 (0.0%)	4 (10.3%)	2 (5.1%)	0 (0.0%)	1 (2.6%)	0.240 **
	Preterm birth	0 (0.0%)	4 (10.3%)	0 (0.0%)	0 (0.0%)	0 (0.0%)	
	term birth	0 (0.0%)	8 (20.5%)	2 (5.1%)	0 (0.0%)	0 (0.0%)	
	Unknown term	5 (12.8%)	8 (20.5%)	4 (10.3%)	1 (2.6%)	0 (0.0%)	
Length	No information recorded	5 (12.5%)	9 (22.5%)	2 (5.0%)	0 (0.0%)	1 (2.5%)	0.012 **
	48–52 cm	0 (0.0%)	14 (35.0%)	2 (5.0%)	0 (0.0%)	0 (0.0%)	
	Under 48 cm	0 (0.0%)	1 (2.5%)	4 (10.0%)	1 (2.5%)	0 (0.0%)	
	Over 52	0 (0.0%)	1 (2.5%)	0 (0.0%)	0 (0.0%)	0 (0.0%)	
APGAR score	No data	5 (12.5%)	6 (15.0%)	0 (0.0%)	0 (0.0%)	1 (2.5%)	0.018 **
	Excellent (10)	0 (0.0%)	2 (5.0%)	1 (2.5%)	0 (0.0%)	0 (0.0%)	
	Good score (7,8,9)	0 (0.0%)	17 (42.5%)	7 (17.5%)	1 (2.5%)	0 (0.0%)	
Jaundice	NO	5 (12.5%)	14 (35.0%)	4 (10.0%)	0 (0.0%)	0 (0.0%)	0.158 **
	FROM	0 (0.0%)	11 (27.5%)	4 (10.0%)	1 (2.5%)	1 (2.5%)	
Newborn feeding	No data	4 (10.0%)	5 (12.5%)	2 (5.0%)	0 (0.0%)	1 (2.5%)	0.643 *
	Breastfed	1 (2.5%)	11 (27.5%)	3 (7.5%)	1 (2.5%)	0 (0.0%)	
	Powdered milk	0 (0.0%)	3 (7.5%)	0 (0.0%)	0 (0.0%)	0 (0.0%)	
	Breastfed and powdered milk	0 (0.0%)	5 (12.5%)	3 (7.5%)	0 (0.0%)	0 (0.0%)	
	Feeding tube + bottle of powdered milk	0 (0.0%)	1 (2.5%)	0 (0.0%)	0 (0.0%)	0 (0.0%)	
Hospitalization history (days)	No data	4 (10.0%)	6 (15.0%)	5 (12.5%)	1 (2.5%)	1 (2.5%)	0.989 *
	1	1 (2.5%)	0 (0.0%)	0 (0.0%)	0 (0.0%)	0 (0.0%)	
	2	0 (0.0%)	1 (2.5%)	0 (0.0%)	0 (0.0%)	0 (0.0%)	
	3	0 (0.0%)	4 (10.0%)	0 (0.0%)	0 (0.0%)	0 (0.0%)	
	4	0 (0.0%)	4 (10.0%)	0 (0.0%)	0 (0.0%)	0 (0.0%)	
	5	0 (0.0%)	1 (2.5%)	0 (0.0%)	0 (0.0%)	0 (0.0%)	
	6	0 (0.0%)	2 (5.0%)	0 (0.0%)	0 (0.0%)	0 (0.0%)	
	7	0 (0.0%)	3 (7.5%)	1 (2.5%)	0 (0.0%)	0 (0.0%)	
	8	0 (0.0%)	1 (2.5%)	0 (0.0%)	0 (0.0%)	0 (0.0%)	
	10	0 (0.0%)	0 (0.0%)	1 (2.5%)	0 (0.0%)	0 (0.0%)	
	11	0 (0.0%)	1 (2.5%)	0 (0.0%)	0 (0.0%)	0 (0.0%)	
	14	0 (0.0%)	0 (0.0%)	1 (2.5%)	0 (0.0%)	0 (0.0%)	
	15	0 (0.0%)	1 (2.5%)	0 (0.0%)	0 (0.0%)	0 (0.0%)	
	21	0 (0.0%)	1 (2.5%)	0 (0.0%)	0 (0.0%)	0 (0.0%)	

* Fisher’s exact test. ** Chi-square test.

**Table 6:** Types of respiratory support administered.

Parameter		Age (Days)				*p*
		Under 14 Days	15–28 Days	28–42 Days	Over 43 Days	
Antibiotics	NO	0 (0.0%)	1 (2.5%)	1 (2.5%)	0 (0.0%)	0.821 **
	YES	8 (20.0%)	12 (30.0%)	14 (35.0%)	4 (10.0%)	
Antiviral/antifungal	NO	8 (20.0%)	12 (30.0%)	14 (35.0%)	3 (7.5%)	0.488 *
	YES	0 (0.0%)	1 (2.5%)	1 (2.5%)	1 (2.5%)	
Corticoids/anti-inflammatories	NO	0 (0.0%)	5 (12.5%)	8 (20.0%)	1 (2.5%)	0.080 *
	YES	8 (20.0%)	8 (20.0%)	7 (17.5%)	3 (7.5%)	
Symptomatic	NO	8 (20.0%)	10 (25.0%)	10 (25.0%)	1 (2.5%)	0.047 *
	YES	0 (0.0%)	3 (7.5%)	5 (12.5%)	3 (7.5%)	
Respiratory support *	NO	1 (2.5%)	4 (10.0%)	6 (15.0%)	0 (0.0%)	0.300 **
	YES	7 (17.5%)	9 (22.5%)	9 (22.5%)	4 (10.0%)	
Gastrointestinal	NO	8 (20.0%)	10 (25.0%)	11 (27.5%)	3 (7.5%)	0.465 *
	YES	0 (0.0%)	3 (7.5%)	4 (10.0%)	1 (2.5%)	
Sedatives/anesthetics	NO	8 (20.0%)	13 (32.5%)	15 (37.5%)	3 (7.5%)	0.026 *
	YES	0 (0.0%)	0 (0.0%)	0 (0.0%)	1 (2.5%)	
Vitamins/supplements	NO	8 (20.0%)	13 (32.5%)	13 (32.5%)	4 (10.0%)	0.320 *
	YES	0 (0.0%)	0 (0.0%)	2 (5.0%)	0 (0.0%)	
Local treatment	NO	6 (15.0%)	10 (25.0%)	8 (20.0%)	3 (7.5%)	0.531 *
	YES	2 (5.0%)	3 (7.5%)	7 (17.5%)	1 (2.5%)	

* Fisher’s exact test. ** Chi-square test.

**Table 7:** N-CRAS structure.

Clinical Variable	Description	Score Awarded
Respiratory infections ^a^	Clinically or paraclinically diagnosed on admission	1 point
Metabolic diseases ^b^	Presence of a documented metabolic pathology	1 point
Symptomatic treatment ^c^	Administered in inpatient care	1 point
Mechanical ventilation ^d^	The need for advanced respiratory support	1 point
Intubation ^e^	The patient was intubated during hospitalization	1 point

^a^ Respiratory infections confirmed by culture or viral panel. ^b^ Metabolic diseases refer to inborn errors of metabolism diagnosed by lab evaluation. ^c^ Symptomatic treatment includes bronchodilators or diuretics. ^d^ Mechanical ventilation defined as invasive ventilation (not CPAP/NIPPV). ^e^ Intubation includes procedures regardless of ventilator connection.

**Table 8:** Distribution of patients by N-CRAS and age.

**Risk Category**	**Age (Days)**			
	**Under 14 Days**	**15–28 Days**	**28–42 Days**	**Over 43 Days**
Low	87.5% (7)	84.6% (11)	66.7% (10)	0.0% (0)
Moderated	12.5% (1)	15.4% (2)	33.3% (5)	50.0% (2)
High	0.0% (0)	0.0% (0)	0.0% (0)	50.0% (2)
Risk Category	Age (Days)			
	Under 14 Days	15–28 Days	28–42 Days	Over 43 Days
Moderated	12.5% (1)	15.4% (2)	33.3% (5)	50.0% (2)
High	0.0% (0)	0.0% (0)	0.0% (0)	50.0% (2)
Low	87.5% (7)	84.6% (11)	66.7% (10)	0.0% (0)

**Table 3:** Distribution of patient history according to maternal history.

Parameter		Monitoring Type				*p*
		No Information	Adequate Prenatal Monitoring	Lack of Prenatal Monitoring	Incomplete Prenatal Monitoring	
Birth type	No information	5 (12.5%)	0 (0.0%)	0 (0.0%)	0 (0.0%)	0.004
	Natural birth	5 (12.5%)	13 (32.5%)	4 (10.0%)	1 (2.5%)	
	Cesarean birth	0 (0.0%)	9 (22.5%)	2 (5.0%)	1 (2.5%)	
Environment of origin	Rural	8 (20.0%)	13 (32.5%)	2 (5.0%)	2 (5.0%)	0.188
	Urban	2 (5.0%)	9 (22.5%)	4 (10.0%)	0 (0.0%)	

The Chi-square test.

**Table 4:** Distribution of perinatal and clinical background characteristics by age group in infants with respiratory symptoms.

Parameter			Age (Days)				*p*
			Under 14 Days	15–28 Days	28–42 Days	Over 43 Days	
Jaundice		NO	4 (10.0%)	6 (15.0%)	11 (27.5%)	2 (5.0%)	0.475 *
		YES	4 (10.0%)	7 (17.5%)	4 (10.0%)	2 (5.0%)	
Incubator		NO	7 (17.5%)	9 (22.5%)	14 (35.0%)	2 (5.0%)	0.162 *
		YES	1 (2.5%)	4 (10.0%)	1 (2.5%)	2 (5.0%)	
Phototherapy		NO	5 (12.8%)	7 (17.9%)	14 (35.9%)	1 (2.6%)	0.057 *
		YES	3 (7.7%)	6 (15.4%)	1 (2.6%)	2 (5.1%)	
Mechanical ventilation		NO	7 (17.5%)	13 (32.5%)	15 (37.5%)	1 (2.5%)	0.008 **
		YES	1 (2.5%)	0 (0.0%)	0 (0.0%)	3 (7.5%)	
Intubated patient		NO	7 (17.5%)	13 (32.5%)	15 (37.5%)	1 (2.5%)	0.008 **
		YES	1 (2.5%)	0 (0.0%)	0 (0.0%)	3 (7.5%)	

* Fisher’s exact test. ** Chi-square test.

**Table 5:** Distribution of current diseases of hospitalized patients by age group.

Parameter		Age (Days)				*p*
		Under 14 Days	15–28 Days	28–42 Days	Over 43 Days	
Respiratory Infections	NO	0 (0.0%)	6 (15.0%)	1 (2.5%)	0 (0.0%)	0.011 **
	YES	8 (20.0%)	7 (17.5%)	14 (35.0%)	4 (10.0%)	
Respiratory Complications	NO	8 (20.0%)	13 (32.5%)	15 (37.5%)	4 (10.0%)	-
Septal Defect	NO	7 (17.5%)	11 (27.5%)	14 (35.0%)	2 (5.0%)	0.355 *
	YES	1 (2.5%)	2 (5.0%)	1 (2.5%)	2 (5.0%)	
Other Heart Conditions	NO	8 (20.0%)	12 (30.0%)	15 (37.5%)	4 (10.0%)	1.000 *
	YES	0 (0.0%)	1 (2.5%)	0 (0.0%)	0 (0.0%)	
Metabolic Diseases	NO	8 (20.0%)	9 (22.5%)	15 (37.5%)	4 (10.0%)	0.026 *
	YES	0 (0.0%)	4 (10.0%)	0 (0.0%)	0 (0.0%)	
Hematologic Diseases	NO	7 (17.5%)	11 (27.5%)	12 (30.0%)	2 (5.0%)	0.439 **
	YES	1 (2.5%)	2 (5.0%)	3 (7.5%)	2 (5.0%)	
Nutritional Diseases	NO	8 (20.0%)	13 (32.5%)	15 (37.5%)	4 (10.0%)	-

* Fisher’s exact test. ** Chi-square test.

## Data Availability

Data are contained within the article.
